# Meaningful use of a digital platform and structured telephone support to facilitate remote person-centred care – a mixed-method study on patient perspectives

**DOI:** 10.1186/s12913-022-07831-8

**Published:** 2022-04-04

**Authors:** Emmelie Barenfeld, Joanne M. Fuller, Sara Wallström, Andreas Fors, Lilas Ali, Inger Ekman

**Affiliations:** 1 Institute of Health and Care Sciences, Sahlgrenska Academy, University of Gothenburg, Box 457, Gothenburg, 40530 Sweden; 2 Centre for Person-Centred Care (GPCC), University of Gothenburg, Gothenburg, Sweden; 3 Department of Occupational Therapy and Physiotherapy, Sahlgrenska University Hospital, Gothenburg, Sweden; 4 Department of Forensic Psychiatry, Sahlgrenska University Hospital, Gothenburg, Sweden; 5 Region Västra Götaland, Research and Development Primary Health Care, Gothenburg, Sweden; 6 Psychiatric Department, Sahlgrenska University Hospital, Gothenburg, Sweden; 7 Department of Medicine, Geriatrics and Emergency Medicine, Sahlgrenska University Hospital/Ostra, Gothenburg, Sweden

**Keywords:** eHealth, Tele-Health, Telemedicine, Patient-centred, Patient-centered, Person-centered, Person-centred, COPD, Chronic heart failure, Chronic condition, Prevention, Process-evaluation

## Abstract

**Background:**

Process evaluations are useful in clarifying results obtained from randomised controlled trials (RCTs). Traditionally, the degree of intervention usage in process evaluations is monitored by measuring dose or evaluating implementation fidelity. From a person-centred perspective, such evaluations should be supplemented with patients’ experiences of meaningful use, given that intervention use should be agreed upon between interested parties and tailored to each patient. This study aimed to elucidate patients’ experiences of a remote person-centred care (PCC) intervention by deepening the understanding of, if, how and for whom the intervention contributed to meaningful use.

**Methods:**

Patients (*n* = 86) were recruited from the RCT PROTECT intervention group. A convergent mixed-method approach was implemented. Data were collected in parallel with the ongoing RCT via a survey, including ratings and written comments on meaningful or non-meaningful use. Also, interviews were performed with twelve purposefully selected participants. Descriptive statistics, logistic regression and content analysis were employed. Data sources were integrated in the results.

**Results:**

Most participants rated the overall intervention as meaningful to use, with the telephone support rated as most meaningful. Interviews and written comments showed that patient ratings on meaningful use were explained by four categories: Not in need, Communication deficiency, Benefits in everyday life and A personal boost*.* Meaningful use of rating symptoms on the digital platform was predicted by living alone (adjusted odds ratio [aOR] = 2.8 *P* = .044). A diagnosis of chronic obstructive pulmonary disease (COPD) predicted meaningful use of digital platform direct messaging (aOR = 3.5, *P* = .045). Moreover, having access to direct-dial telephone contact explained meaningful use among participants with low ratings of technical competence (aOR = 3.6, *P* = .014).

**Conclusions:**

The combined digital platform and structured telephone support could be helpful in identifying preventive actions to maintain health for people diagnosed with COPD and chronic heart failure but tends to be more meaningful for those diagnosed with COPD. Overall, lower adoption of the digital platform was seen compared to telephone support. Shortcomings were noted in the digital platform’s implementation that negatively influences experiences of meaningful use. When used, the intervention proved to be an easily applicable and valued tool to support preventive actions in a person-centred manner.

## Background

A call for easily accessible health services is considered important to support self-management activities for most people living with chronic conditions, such as chronic obstructive pulmonary disease (COPD) and chronic heart failure (CHF) [1]. Person-centred care (PCC) and remote interventions (e.g., telehealth and e-health solutions) are essential in such service process redesign in health care [2, 3]. From a person-centred standpoint, all care should support people in taking responsibility for their health [4], facilitate narration, provide transparent documentation of shared decision making and offer the opportunity to work in partnership with health care professionals and family members [5]. These dimensions are central to the person-centred approach applied to the remote PCC intervention [6] explored in this study.

To date, little is known about how the design and content of remote interventions influence patients’ opportunities to work in partnership. A review [7] on PCC performed remotely found that very few interventions were fully person-centred. While studies exist on adoption [8, 9] and experiences of using remote interventions by patients with COPD or CHF [10, 11], there remains a knowledge gap on person-centred aspects (e.g., how shared decision making, personal information sharing and setting up a health plan are implemented remotely) [7].

Process evaluations are needed to complement randomised controlled trials (RCTs) and measure how patients’ interactions with complex interventions and contextual influences may explain implementation or intervention outcomes [12]. Intervention use and factors affecting its use are important outputs in process evaluations as they provide details on received intervention dose and what might influence use [13]. Traditionally, the degree of intervention usage in process evaluations is monitored by measuring dose or evaluating implementation fidelity (the degree to which an intervention is delivered as intended) [12, 14]. From a person-centred perspective, there is a need to complement process evaluations with patients’ experiences of the meaningful use of interventions, as intervention use should be agreed upon between patients and practitioners and tailored to each patient [4]. In this article, the term meaningful use is applied to capture both purposes and actions performed by patients to aim for health through partnership using a remote PCC intervention.

The remote PCC intervention (being a combined digital platform and structured telephone support for people with COPD and CHF) [6] has shown potential in facilitating interaction between self-management and working in partnership [15]. However, patients’ experiences of facilitators and hindrances of using such person-centred remote interventions merit further exploration. Thus, this study aimed to elucidate patients’ experiences of a remote PCC intervention by deepening the understanding of if, how and for whom the support contributed to meaningful use.

Specifically, three research questions were formulated:

1. *Do patients experience that the intervention is meaningful to use?*
2. *Are there patient characteristics or intervention functions that explain patients´ reports of meaningful or non-meaningful use?*
3. *How do patients describe their experiences of meaningful and non-meaningful use?*

## Methods

### Design

A convergent parallel mixed-method design [16, 17] was applied. This design enables quantitative results to be illustrated by qualitative findings.

### Study setting and the remote PCC intervention

This study is a part of a process evaluation of the RCT trial PROTECT (NCT03183817). Patients diagnosed with COPD and CHF listed in nine primary care centres in Sweden were included in the study. The intervention group in the PROTECT trial received remote PCC through a digital platform and structured telephone support as an add-on-treatment to usual care during the study period (six months). The intervention is described in detail elsewhere [6, 15]. Briefly, the intervention aimed to operationalise person-centred ethics into action by safeguarding the relational aspects of personhood in care relying on an evidence-based PCC approach [4, 18]. The support was designed to facilitate partnership between professionals and patients and also the patients’ families and friends. Structured telephone and digital platform support functions and working methods stimulate patients’ narration and agreement in health planning. Moreover, the digital platform was used to achieve shared documentation and transparency in the partnership. Health professionals representing different disciplines (nursing, physiotherapy, and occupational therapy) provided the intervention. See Table 1 for an overview of functions integrated into the intervention.

**Table 1:** Overview of functions in the remote PCC intervention

Co-creation and follow-up of tailored health plans via structured telephone support
Access to their health plans on the digital platform
Writing their health plan
Invitation to family and friends to the digital platform
Rating symptoms and wellbeing and monitoring trend graphs of self-ratings
Daily access to health professionals via structured telephone support or the digital platform
Validated links about CHF and COPD and other useful health information sites

### Participants

Participants were recruited from the intervention group in the PROTECT trial (*n* = 110). A run-in period was used to distribute the process evaluation questionnaire, which resulted in that the first 14 participants were excluded from this study. A further 10 participants received the questionnaire but did not respond. Thus, the final sample of 86 participants was included in this process evaluation.

### Data sources and collection

Data from quantitative and qualitative approaches were collected in parallel. The two approaches were given equal priority in data collection and analysis (Table 2).

**Table 2:** Overview of data sources, data collection procedures and analysis in the quantitative and qualitative approaches

	Quantitative approach	Qualitative approach	
**Data source**	Questionnaire *n* = 86	Written comments *n* = 44	Interviews *n* = 12
**Inclusion criteria**	Participated in the PROTECT trial intervention group		Purposeful sampling to capture heterogeneity across age, sex, educational level and e-support use
	Diagnosed with COPD or CHF		
	Ability to understand written and spoken Swedish		
	Listed at one of the nine participating primary care centres in the PROTECT-trial		
**Data collection**	Self-ratings	Written comments	Face-to-face or telephone interviews
**Questions**	Three questions	One question	One opening question
	Demographic questions		Interview guide with question areas
			Probes
**Data analysis**	Descriptive statistics and logistic regression analyses	Content analysis	Content analysis

*CHF* Chronic Heart Failure, *COPD* Chronic Obstructive Pulmonary Disease, PROTECT = name of the evaluated remote PCC intervention

#### Questionnaires and procedures

Patient characteristics and demographics were collected through the questionnaire included in the RCT trial and from medical records. The process evaluation questionnaire was distributed by post following intervention completion. The questionnaire was developed for this study and collected ratings of the meaningful use of the overall intervention and meaningful functions included in the intervention (see Table 1), as well as the technical competence of the participants in the intervention group. Three questions explored these areas:

1. *How well does the following statement fit into your experience of using the combined digital platform and structured telephone support: I feel that the remote support has been meaningful to me (Agree, Partly agree, Partly disagree, Disagree, Don´t know).* This question was followed by the opportunity to provide a comment (question 1b) to describe *in what way the combined digital platform and structured telephone support have been meaningful or not meaningful to use****.***
2. *Which functions in the combined digital platform and structured telephone support have been meaningful to you?*
3. *How do you perceive your technology skills, i.e., your ability to use technical equipment such as a smartphone, digital tablet or computer (Excellent, Very good, Good, Fair, Bad)?*

#### Individual interviews

After the completion of the intervention, the first author (EB) a registered occupational therapist and researcher within the field of PCC and health promotion experienced in qualitative research performed individual interviews with 12 patients. The first author had no established relationship with the participants prior to the study. Participants were encouraged to tell as candid and detailed as possible about their experiences of using the remote intervention. Interviews were performed with the support of an interview guide designed to collect data for several process evaluation sub-studies in the PROTECT trial. Further details on individual interviews and the interview guide are described elsewhere (see Table 2 for an overview) [15].

### Analysis

#### Statistical analysis

Continuous variables are described by mean, standard deviation, median, minimum, and maximum and categorical variables by numbers and percentages. For comparison between two groups, the t-test was used for continuous variables, Pearson’s chi-square test for categorical variables and Fisher's exact test for dichotomous variables. Predictors of meaningful use were analysed by univariable and stepwise multivariable logistic regression and results given as odds ratios (ORs) respectively adjusted odds ratios (aORs) with 95% confidence intervals (CIs) and area under the ROC curve (AUC). Participants diagnosed with CHF and COPD (*n* = 7) were excluded from multiple logistic regression analysis due to the small group size. All statistical tests were two-sided, and the significance level was set at *P* < 0.05.

Dependent variables were the meaningful use of the remote PCC intervention and specific intervention functions (see Table 1). Meaningful use of the overall intervention was dichotomised into yes (Fully agree, Partly agree) or no (Partly disagree, Disagree, Don´t know) answers. The ‘yes’ responses were considered as meaningful use and ‘no’ responses as non-meaningful use.

Independent variables were diagnosis (CHF, COPD or both), civil status, sex, age, education level (compulsory or lower, secondary school, vocational college, university) and self-rated technical competence (dichotomised as good [Excellent, Very good, Good] and poor [Fair, Bad]).

#### Analysis of qualitative data sources

The qualitative analysis was performed in two steps. First, a conventional content analysis was conducted of the written comments [19]. The replies from the question “*Describe in what way the combined digital platform and structured telephone support has been meaningful or not meaningful to use”* were divided into two groups representing ratings on meaningful and non-meaningful use. The comments of meaningful and non-meaningful use were read several times to obtain a sense of the whole. After that, meaning units were identified, condensed, and coded. The codes were compared for similarities and differences. Codes with similar content were grouped into categories [19], which resulted in five preliminary categories.

Second, the five preliminary categories based on the written comments were used as a framework in directed content analysis [19]. Twelve individual interviews were added to the analysis to deepen the understanding of the preliminary categories. Interviews were coded and codes were sorted into one of the five categories. This iterative process involved developing sub-categories and further developing categories (e.g., converting one of the preliminary categories to a sub-category and re-formulating category names). The first author (EB) performed the main analyses and was responsible for coding the data. To establish trustworthiness, co-authors with different disciplines and gender verified the analyses of the written comments (AF, IE) and individual interviews (JF, LA, AF, IE). All authors agreed upon the final qualitative analysis containing four categories and 12 sub-categories. N-Vivo 12 was used to store and organise the data.

#### Integration of quantitative and qualitative findings

Statistical analysis of self-ratings and analysis of written comments and interviews were performed separately. After that, data from quantitative and qualitative data sources were synthesised during the interpretation phase. The integration took place in the presentation of results.

### Ethics approval and consent to participate

The study was approved by the Regional Ethical Review Board located in Gothenburg, Sweden (063–17 and T613-18). We confirm that all methods were performed in accordance with the relevant guidelines and regulations. Written informed consent was obtained from all participants.

## Results

### Patient characteristics

Data from 86 patients were included in this process evaluation, of which 44 people contributed to both qualitative and quantitative data collection. An overview of the participant characteristics and demographics is shown in Table 3.

**Table 3:** Participant characteristics and demographics

	Participants included in quantitative process evaluation *n* = 86	Not included *n* = 24	*P*-value	Participants providing written comments *n* = 44	Interviewees *n* = 12
**Age**					
Mean, years (SD)	71.3 (9.2)	70.2 (11.7)	.63	71.8 (10.0)	71.4
Median, years (min, max)	72.5 (33–93)	71.0 (42–90)		72.5 (33–93)	73 (57–81)
**Sex**					
Women (%)	40 (46.5%)	11 (45.8%)	1.00	24 (54.5%)	5 (41.7%)
**Civil status**					
Living alone (%)	36 (41.9%)	6 (25%)	0.22	18 (40.9%)	2 (16.7%)
**Diagnosis**					
CHF (%)	34 (39.5%)	8 (33.3%)	0.21	12 (27.3%)	1 (8.3%)
COPD (%)	45 (52.3%)	11 (45.8%)		28 (63.6%)	9 (75.0%)
CHF and COPD (%)	7 (8.1%)	5 (20.8%)		4 (9.1%)	2 (16.7%)
**Education level**					
Compulsory (%)	28 (32.6%)	10 (41.7%)	0.24	14 (3.,8%)	4 (33.3%)
Secondary school (%)	17 (19.8%)	8 (33.3%)		9 (20.5%)	4 (33.3%)
Vocational college (%)	22 (25.6%)	3 (12.5%)		10 (22.7%)	1 (8.3%)
University (%)	19 (22.1%)	3 (12.5%)		11 (25.0%)	3 (25.0%)
**Self-rated technical competence**					
Good or better (%)	51 (60,0%)	6^a^ (40%)	0.22	27 (61.4%)	9 (75,0%)
**Use of intervention functions**					
Number of phone calls (median, min–max)	3, (0–6)	3, (0–4)	1.00	4, (1–6)	3, (2–5)
Used digital platform functions (%)	60 (69.8%)	16 (66.7%)	0.81	32 (72.7%)	11 (91.7%)

^a^missing *n* = 10

*CHF* Chronic Heart Failure, *COPD* Chronic Obstructive Pulmonary Disease, *SD* Standard Deviation

### Was the intervention meaningful?

Overall, 63.9% of the participants rated the combined digital platform and telephone support as meaningful to use, 14.5% rated it as non-meaningful and 21.7% answered that they did not know. The intervention component rated as most meaningful by most of the participants was telephone support, followed by being able to rate your daily health and having the possibility to contact health care professionals with a direct telephone number or by direct messaging through the digital platform (Table 4). Only one participant reported that the function of inviting family and friends to the digital platform was meaningful to use.

**Table 4:** Overview of ratings on meaningful use for the overall intervention and specific intervention functions, *n* = 86

	Overall intervention	Structured telephone support		Digital platform functions					
		Phone calls	Direct-dial contact	Ratings	Follow trend graphs	Direct messaging	Access to health plan	Write own health plan	Validated CHF & COPD links
**Total sample** *n* = 86	53(63.9%)^a^	58(69.9%)^a^	26(31.3%)^a^	26(31.3%)^a^	8(9.6%)^a^	18(21.7%)^a^	12(14.5%)^a^	6(7.2%)^a^	13(15.7%)^a^
**Diagnosis**									
CHF *n* = 34	18(58.1%)^a^	21(63.6%)^b^	10(30.3%)^b^	9(27.3%)^b^	1(3.0%)^b^	4(12.1%)^b^	3(9.1%)^b^	2(6.1%)^b^	2(6.1%)^b^
COPD *n* = 45	34(75.6%)	33(76.7%)^c^	13(30.2%)^c^	16 (37.2%)^c^	4(14.0%)^c^	14(32.6%)^c^	9(20.9%)^c^	4(9.3%)^c^	8(18.6%)^c^
CHF & COPD *n* = 7	1(14.3%)	4(57.1%)	0(0.0%)	1(14.3%)	1(14.3%)	0(0.0%)	0(0.0%)	0(0.0%)	3(42.9%)
**Civil status**									
Living alone *n* = 36	22(66.7%)^a^	27(79.4%)^b^	12(35.3%)^b^	16(47.1%)^b^	5(14.7%)^b^	11(32.4%)^b^	8(23.5%)^b^	4(11.8%)^b^	7(20.6%)^b^
Married/Partner *n* = 50	31(62.0%)	31(63.3%)^c^	11(22.4%)^c^	10(20.4%)^c^	3(6.1%)^c^	7(14.3%)^c^	4(8.2%)^c^	2(4.1%)^c^	6(12.2%)^c^
**Sex**									
Male *n* = 46	26(59.1%)^c^	29(64.4%)^b^	7(15.6%)^b^	14(31.1%)^b^	4(8.9%)^b^	7(15.6%)^b^	5(11.1%)^b^	3(6.7%)^b^	8(17.8%)^b^
Female *n* = 40	27(69.2%)^b^	29(76.3%)^c^	16(42.1%)^c^	12(31.6%)^c^	4(10.5%)^c^	11(28.9%)^c^	7(18.4%)^c^	3(7.9%)^c^	5(13.2%)^c^
**Technical Competence**^d^									
Good *n* = 51	34(66.7%)	34(68.0%)^b^	9(18.0%)^b^	15(30.0%)^b^	5(10.0%)^b^	12(24.0%)^b^	8(16.0%)^b^	6(12.0%)^b^	10(20.0%)^b^
Poor *n* = 34	19(61.3%)^a^	23(71.9%)^c^	13(40.6%)^c^	10(29.0%)^c^	3(9.4%)^c^	6(18.8%)^c^	3(9.4%)^c^	0(0.0%)^c^	3(9,4%)^c^
**Educational level**									
< Secondary *n* = 45	30(71.4%)^a^	27(67.5%)^c^	11(25.6%)^c^	12(27.9%)^c^	4(9.3%)^c^	11(25,6%)^c^	7(16,3%)^c^	1(2,3%)^c^	6(14,0%)^c^
> Secondary *n* = 41	23(56,1%)	31(72,1%)^b^	12(30,0%)^b^	14(35,0%)^b^	4(10%)^b^	7(17.5%)^b^	5(12.5%)^b^	5(12.5%)^b^	7(17.5%)^b^
**Age**									
< 74 years *n* = 56	39(69.9%)	39(70.9%)^b^	15(27.3%)^b^	16(29.1%)^b^	3(5.5%)^b^	13(23.6%)^b^	8(14.5%) ^b^	5(9.1%)^b^	10(18.2%)^b^
> 75 years *n* = 30	14(51.9%)^a^	19(67.9%)^c^	8(28.6%)^c^	10(35.7%)^c^	5(17.9%)^c^	5(17.9%)^c^	4(14.3%)^c^	1(3,6%)^c^	3(10.7%)^c^

^a^missing = 3, ^b^missing = 1, ^c^missing = 2, ^d^*n* = 85

*CHF* Chronic Heart Failure, *COPD* Chronic Obstructive Pulmonary Disease

### Who found the intervention meaningful?

Participants with COPD showed a trend in rating the remote PCC intervention and its functions as more meaningful to use compared to people diagnosed with CHF and the total sample. This trend was also observed among participants living alone. Moreover, the proportion of participants rating the intervention and its different functions as meaningful was lower among people diagnosed with both CHF and COPD than those with one of the diagnoses, except for the function of using validated links (see Table 4).

Logistic regression was performed to assess the likelihood of patient characteristics to explain the overall meaningful use of the intervention and the four most appreciated intervention functions (Table 5). None of the explored patient characteristics (diagnosis, civil status, sex, education level, technical competence, and age) could significantly predict the overall meaningful use of the intervention or on person-centred dialogues by way of structured telephone support. Some patient characteristics (diagnosis, civil status, sex, and technical competence) could significantly predict specific intervention functions (Table 4). Stepwise multiple logistic regression identified which factors to include in the models most likely to predict meaningful use per intervention function (Table 6). All final models included one predictor. Rating technical skills as poor predicted the meaningful use of a direct telephone number to health professionals (aOR = 3.6, *P* = 0.014). COPD diagnosis predicted ratings on meaningful use to contact professionals by direct messaging through the digital platform (aOR = 3.5, *P* = 0.045). Living alone was a significant predictor of symptoms and daily health ratings on the digital platform as meaningful to use (aOR = 2.8 *P* = 0.044).

**Table 5:** Meaningful use per patient characteristic and reports of odds ratios *n* = 86

	OR (95% CI)	P-value	AUC
**Intervention overall**			
COPD *n* = 76	2.2 (0.8 -6.0)	.11	0.60
Living alone *n* = 83	1.2 (0.5 -3.1)	.67	0.52
Women *n* = 83	1.6 (0.6 -3.9)	.34	0.56
Technical skills rated as poor *n* = 82	0.8 (0.3 -2.0)	.62	0.53
Education/level *n* = 83	0.7 (0.5 -1.1)	.087	0.61
Age/5 year *n* = 83	0.9 (0.7 -1.2)	.58	0.55
**Person-centred dialogue**			
COPD *n* = 76	1.9 (0.7 -5.1)	.22	0,58
Living alone *n* = 83	2.2 (0.8 -6.2)	.12	0,59
Women *n* = 83	1.8 (0.7 -4.7)	.24	0,57
Technical skills rated as poor *n* = 82	1.3 (0.5 -3.3)	.65	0,53
Education/level *n* = 83	0.8 (0,5 -1,1)	.19	0,59
Age/5 year *n* = 83	0.9 (0.7 -1.2)	.59	0,54
**Contact possibility via phone**			
COPD *n* = 76	1.0 (0.4 -2.7)	.99	0,50
Living alone *n* = 83	1.9 (0.7 -5.0)	.20	0,58
Women *n* = 83	3.9 (1.4 -11.1)	.009	0,66
Technical skills rated as poor *n* = 82	3.4 (1.2 -9.1)	.017	0,65
Education/level *n* = 83	1.0 (0.6 -1.5)	.85	0,51
Age/5 year *n* = 83	1.0 (0.8 -1.3)	.90	0,49
**Direct messaging on platform**			
COPD *n* = 76	3.5 (1.03 -11.9)	.045	0,64
Living alone *n* = 83	2.9 (0.98 -8.4)	.055	0,63
Women *n* = 83	2.2 (0.8 -6.4)	.15	0,60
Technical skills rated as poor *n* = 82	0.7 (0.2 -2.1)	.53	0,54
Education/level *n* = 83	0.8 (0.5 -1.2)	.26	0,58
Age/5 year *n* = 83	1 (0.8 -1.4)	.76	0,50
**Ratings on digital platform**			
COPD *n* = 76	1.6 (0.6 -4.2)	.36	0,56
Living alone *n* = 83	3.5 (1.3 -9.1)	.012	0,65
Women *n* = 83	1 (0.4 -2.6)	.96	0,50
Technical skills rated as poor *n* = 82	1.2 (0.5 -3)	.75	0,52
Education/level *n* = 83	1.1 (0.7 -1.6)	.69	0,53
Age/5 year *n* = 83	1 (0.8 -1.3)	.84	0,53

*AUC* area under the ROC curve, *CI* confidence interval, *OR* odds ratio

**Table 6:** Predictors included in the multiple regression-models most likely to explain meaningful use, *n* = 79

Predictors per intervention function		*P*-value	Adjusted OR (95% CI)	AUC
Intervention function	Predictor			
**Direct dial-in telephone support**	Rating technical competence as poor	.014	3.6 (1.3–10.0)	0.65
**Direct messaging via the digital platform**	Diagnosis of COPD	.045	3.5 (1,03–11,9)	0.64
**Ratings in the digital platform**	Living alone	.044	2.8 (1.03–7.4)	0.62

*AUC* area under the ROC curve, *CI* confidence interval, *OR* odds ratio

### How is meaningful and non-meaningful use explained?

The integrated analysis on self-ratings with written comments and individual interviews resulted in four categories and 12 sub-categories identified to explain patients’ ratings of meaningful use. Non-meaningful use was described in two categories: Not in need and Deficiency in communication. Meaningful use was also formulated in two categories: Benefits in everyday life and A personal boost (see Table 7 for an overview of the categories and sub-categories). Quotes from both written comments and the individual interviews are provided in the results section to illustrate explanations of the ratings.

**Table 7:** Overview of the categories and sub-categories by meaningful and non-meaningful use

	Non-meaningful use^a^		Meaningful use^a^	
**Categories**	Not in need	Deficiency in communication	Benefits in everyday life	A personal boost
**Sub-categories**	Health under control	Issues with technology inhibits contact	Feeling safe and secure	Being met with a personal commitment
	Adequate care support	Lacking personal contact	Flexible use of time and reduced stress	Seeing what I need and want
		Unexpressed aims and expectations	Measure of health	Setting a common course of action
				Finding strength for the next step

^a^Overall rating of the remote PCC intervention as meaningful to use dichotomised into yes and no answers

### Factors contributing non-meaningful use

#### Not in need

The Not in need category adheres to the ‘Non-meaningful use’ or ‘Do not know’ answers. The category shows that the remote PCC intervention was not perceived as beneficial for patients to use, but could suit people in need of support, including themselves, later. Two sub-categories were identified to explain if in need or not, namely Health under control and Adequate care support.

##### Health under control

Health under control means that patients did not currently need to use the remote PCC intervention as they managed their everyday lives and reported a high level of wellbeing. Health concerns varied over time and although the remote PCC intervention was not perceived as meaningful, the potential for later use was raised. One example of this was provided in a written comment by a participant diagnosed with COPD:

*Because I haven’t needed much help at the moment, I feel fine and don’t feel limited. It serves no useful purpose. If I were in a difficult situation, I could imagine this being a good tool.*

##### Adequate care support

Adequate care support relates to a need for remote PCC intervention in patients who lacked functioning relationships and care support. *"I’ve had very good contact with my ordinary health care professionals, so I haven’t needed to communicate with anybody (*via* the remote PCC support).”* The period directly after diagnosis of COPD or CHF was associated with a need to use the intervention. This need was particularly emphasised by patients diagnosed with COPD who often experienced a lack of personal follow-up.

#### Deficiency in communication

Deficiency in communication is associated with negative experiences of using the remote PCC intervention as it constituted a barrier to effective communication between patients and staff. Three sub-categories were identified: Issues with technology inhibits contact, Lacking personal contact and Unexpressed aims and expectations.

##### Issues with technology inhibits contact

Issues with technology inhibits contact means that the digital platform inhibited communication because of logging in or connectivity issues. Novice technology users emphasised the importance of receiving training and support in their learning process, which bridged obstacles to digital platform use.

*I had to phone (explains). I couldn’t even remember my login. But then she (staff member) said, “Oh, I’ve got it here.” And I was so happy, and then you could save it (password), so I didn’t have to write it all down. So it went well”.*

##### Lacking personal contact

Lacking personal contact indicates that the physical distance between staff and patients was an obstacle to building relationships. The category refers to occasions when patients did not get the response they expected from the staff, as expressed in the following written comment, *"Never hear from you."* It also referred to participants longing for face-to-face meetings with professionals, as told by a participant diagnosed with COPD in an interview.

*I think it’s nice if you can meet and talk [if possible]. It’s a benefit if you are (in a face-to-face meeting). When you meet in person, you feel like they care.*

##### Unexpressed aims and expectations

Unexpressed aims and expectations signify that professionals and patients did not always clearly communicate their thoughts and expectations using the remote PCC intervention. The participants found it hard to understand the benefits of using the remote support platform and what roles they, their family and staff had in using it. “*I don’t understand what it (the remote PCC support platform) is supposed to be used for.”* Aims and expectations could be clarified over time, which allowed patients to find new purposes in using the intervention or finding alternative ways to use it.

### Factors promoting meaningful use

#### Benefits in everyday life

The category Benefits in everyday life is linked to experiences of the remote PCC intervention as partially or fully meaningful to use. Three sub-categories were identified that described different types of benefit: Feeling safe and secure, Flexible use of time and reduced stress and Measure of health.

##### Feeling safe and secure

Feeling safe and secure entails that the remote PCC intervention contributed to patients’ experiences of a sense of security by being able to rely on trustworthy and competent professional contact. Knowing that there was a direct number to telephone support if needed or an opportunity to get in touch through messaging on the digital platform led to a secure feeling. *“The thing that meant most to me was knowing that she [the nurse] was there.”*

##### Flexible use of time and reduced stress

Flexible use of time and reduced stress entails patients’ experiences of communicating with the remote PCC support platform when they please or from different locations (e.g., at home or abroad). The remote intervention also reduced apprehension that would normally trigger patients’ feeling stressed and concerned about time (e.g., staff showing that they are short of time or stressful expressions of other patients in the waiting room). Thus, this allows them to feel confident in taking their time to ask questions or initiate conversations as stated in the interview:

*It becomes another type of conversation about the subject. If the doctor had time-pressure [in a physical meeting], you would sense it as a patient. You don’t feel that stress in the same way with this remote support [tool].*

##### Measure of health

Measure of health is about the patients’ perceived benefit of receiving answers about their health and illness. The patients also experienced that they were given guidance on when they needed to seek professional support. Telephone conversations, health plans and self-ratings could help identify health decline and improvement, as well as an overview of changes over time. One woman expressed how the rating of daily symptoms helped to recognise her innate capabilities in a period of health decline:

*It has been great [with rating my health]. Because now I am able to perceive for about one week now that I was quite alert and so on [she describes that she thought it was worse than it was]. It was a way for me to see and ascertain [my own health state]. You not only felt bad but you could see that something was also positive.*

#### A personal boost

The category A personal boost is linked to ratings of partially or completely meaningful use of the remote PCC intervention. The category denotes experiences of personally tailored support facilitating health planning and taking action, stimulating participants' reflections on health and personal growth. Four sub-categories were identified: Being met with personal commitment, Seeing what I need and want, Setting a common course to follow and Finding strength for the next step.

##### Being met with a personal commitment

Being met with a personal commitment refers to meeting professionals that were present in the moment and showed genuine care. The patients reported situations where they felt listened to and could share their stories without feeling judged or questioned. A woman diagnosed with COPD provided the following written comment:

*It was the first time that I talked about my COPD and that someone was willing to listen and support me. In this way it was meaningful. That someone cared. Because I have felt “alone” with my COPD, so this has given me so much. Otherwise, it’s been a spirometry test once a year and no actual engagement.*

##### Seeing what I need and want

Seeing what I need and want shows that the remote PCC intervention helped make visible patients’ circumstances and prerequisites in managing their health and creating awareness of what health meant for them. Patients’ formulating their health plan and other activities on the platform motivated them to put their thoughts into words, think about their mood and visualise their current health and how it changed over time. Through conversations with staff, the patients were supported in realising what they needed, wanted, and could do to improve their health and wellness. In an interview, a participant diagnosed with COPD said, “*I have become somewhat more aware of what I want to do and how to get there. “*

##### Setting a common course to action

Setting a common course to action refers to how patients and staff focus on the ways health could be maintained or improved. This collaboration implies that the patients had a shared understanding and that it was clear how they could use the intervention in their daily life to promote health. Patients who wanted to improve their situation emphasised the importance of brainstorming and sharing their thoughts with a professional. Telephone conversations, health plans and self-ratings helped support this process of following a common course. A woman diagnosed with CHF wrote, *“When I had contact plus used the support [tool], I was very sick and the personal contact plus health goals (written) meant that I felt supported and could follow my process”.*

##### Finding strength for the next step

Finding strength for the next step is about the remote PCC intervention contributing to moving further towards to goal of attaining good health by motivating, providing energy, confirmation, and support. Thus, the intervention became a source of support in initiating new actions and as a motivator to continue with what had already been done to feel good. One participant said this about the intervention:

*This and this and this is what I want to do – Oh that’s good (!), that’s good (!) (mimics staff member’s voice). I mean, I already had those thoughts (about what I wanted to do) without having said them to anybody, so to speak, but it’s really good to be encouraged. That yes, it is good. Do it. It’s that bit that you get help with.*

## Discussion

### Main findings

This study aimed to elucidate patients’ experiences of a remote PCC intervention (through a combined digital platform and structured telephone support) by deepening the understanding of, if, how and for whom the support contributed to meaningful use. The intervention proved to be an easily applicable and valued tool to support preventive measures in a person-centred manner. Most participants rated the overall intervention as meaningful to use. The intervention tended to be more meaningful for those diagnosed with COPD compared to those diagnosed with CHF. The main finding is that the structured telephone support contributed to a meaningful use of the intervention by most of the participants. The digital platform was reported as less meaningful to use by the majority of the participants, but some participants appreciated it, especially those with a COPD diagnosis or those living alone. Not being in need of support and deficiency in communication are circumstances which explained ratings of non-meaningful use whereas seeing benefits in everyday life or gaining a personal boost when engaging in the intervention contribute to meaningful use.

### Comparison with prior work

From a patient perspective, our findings deepen understanding of how, when and for whom a remote PCC intervention is meaningful to use. Our findings help clarify which aspects facilitate or hinder such use in supporting self-management of chronic conditions. Research that describes how remote support is provided to and experienced from the patient perspective is generally lacking particularly interventions that involve PCC [7, 20]. A limited number of studies have explored patients’ perspectives on their use of and engagement in remote interventions [10, 21, 22], and few studies have had PCC as their main theoretical basis [15, 23]. Thus, our findings provide key knowledge to support the future development and implementation of remote PCC which, to date, remains an under-investigated area [7, 20].

The structured telephone support seems to play a crucial role in contributing to meaningful use by facilitating the initiation of partnership and agreement of a health plan between health professionals and patients. The category A personal boost and its subcategories provide information on four intervention mechanisms which contributed to meaningful use by supporting health-planning and taking action (Being met with personal commitment, Seeing what I need and want, Setting a common course to follow and Finding strength for the next step). These four intervention mechanisms were predominantly initiated by the structured telephone support, and for some participants also promoted by the digital support. The identified intervention mechanisms are consistent with person-centred processes described in the literature [24, 25], and support Håkansson and co-workers’ [26] review on differences between patient- and person-centred interventions which concludes that PCC aimed at a meaningful life and patient-centred care at achieving functions. In the telehealth context, one RCT evaluating telephone support for people with COPD or CHF concluded that PCC could be delivered remotely but did not describe the process of how PCC was realised and if it was reported as meaningful by users [27]. Our study gives a practical example of how PCC could be applied remotely, and that the initiation of person-centred processes contributed to meaningful use.

Intervention mechanisms that enable relationships with health professionals and visualise symptoms to enhance self-awareness among patients have been reported as facilitators when implementing remote health care interventions [9]. The category Benefits in everyday life explained how both the digital platform and telephone support contributed to meaningful use by having easy access to a trustworthy professional contact and experiencing reduced feelings of stress which enabled dialogues where patients felt welcomed to initiate conversations and ask questions. This category also illustrated how documentation through self-ratings and health plans on the digital platform contributed to meaningful use by identifying both health resources and obstacles to maintaining health. Documentation transparency by providing access to shared health-planning and health-ratings via digital supports is described as a potential contributor to increase patient involvement [5, 28] and is also highlighted to meet the minimum requirement of PCC [25]. However, our results show that few participants rated the function of having access to the documentation of their health plan via the digital platform as meaningful. In contrast, the analysis of written comments and interviews show that meaningful use of the intervention was explained by the way the health plan was co-created and that the intervention provided prerequisites to mutual dialogues. One explanation for this finding could be that informal aspects of the partnership (e.g., personal contact and verbal agreements on the next step) are more important to patients then the formal aspects such as documentation of health planning. Our finding is congruent with a previous qualitative study [29] showing that patients valued actions leading to human connectedness above formalised aspects, although it also showed the value of having a documented health plan for some participants.

Our analysis also revealed barriers to meaningful use related to working remotely in general and specifically for person-centred practices. Deficiencies in communication and its sub-categories (Issues with technology inhibits contact, Lacking personal contact, Unexpressed aims and expectations) clarify which factors inhibited meaningful use and challenged PCC implementation. These findings confirm previous reports on limited access as the main obstacle to digital platform usage [30] and the lack of face-to-face contact in remote interventions [31, 32]. Our qualitative findings on communication barriers were congruent with our quantitative findings, showing a lower adoption of digital platform use than telephone support. Almost one in three participants had not used the digital platform and thus did not receive the intervention according to protocol [6].

The sub-category Unexpressed aims and expectations demonstrate that it was not always clear to patients how the remote PCC intervention could be used or its purpose. This lack of clarity could partially explain the low ratings of meaningful use on some of the digital platform functions (i.e., having shared access to health plans and following trend graphs of self-ratings). However, when applied, these digital platform functions were also described as key elements contributing to meaningful use as reported in our qualitative findings (Benefits in everyday life and A personal boost).

When timed well, the remote PCC intervention could be helpful to identify and support preventive actions to maintain health measure in people diagnosed with COPD or CHF [15]. However, our findings pinpoint people diagnosed with COPD and people living alone as target groups who find the support more meaningful to use. A possible explanation for higher ratings of meaningful use among people diagnosed with COPD could be that they have a greater need to use the remote PCC intervention because of a lack of support in conventional care [5]. This explanation is compatible with our qualitative findings on what contributes to meaningful use and the category “Not in need,” which reported experiences of non-meaningful use due to having Health under control or already experiencing Adequate care support. Outpatient heart failure clinics have been expanded during the past 10 years [33], whereas health care is not as well organised to support people with COPD to the same extent [5, 34]. Studies have also shown that remote interventions for people diagnosed with COPD should emphasise work in partnership, as a diagnosis of COPD is often associated with stigma and shame [5, 34, 35]. Our findings show that the remote PCC intervention was an easily applicable tool to bridge inequities in access to preventive care.

### Limitations

The analysis was derived from responses on ratings (*n* = 86) and comments (*n* = 44) on the questionnaire’s open-ended question as well as individual interviews, making it possible to integrate quantitative and qualitative findings, which strengthens the study’s credibility as the findings from both types of data collection methods confirm the results of the other [17]. One study limitation was the delay in distributing the process evaluation questionnaires that excluded the first 14 participants in the RCT intervention group. However, despite this limitation, the response rate in the process evaluation on quantitative measures was high (75%). The participants' characteristics and demographics were representative when compared to the intervention group as a whole (Table 3). Inclusion criteria for the interview study may have resulted in the participants following the intervention protocol more closely than the process evaluation group, which may have impacted the findings by providing more details on intervention use from people keen to use the remote PCC intervention. However, this fact should not only be regarded as a limitation but also as a strength because interviewing people with experiences of using the support provided explanations of both non-meaningful and meaningful use. Qualitative data was also collected from written comments covering explanations from both non-users and users of the remote PCC intervention which contributed different perspectives on meaningful use. After adding 12 individual interviews to deepen the analysis of preliminary categories developed from the written comments, no new patterns emerged in category development as categories and sub-categories were replicated, thus data was considered saturated [36]. Although PCC was a key-component in the design, we did not explicitly ask participants of how PCC contributed meaningful use as our aim was to evaluate the overall intervention. Future studies could benefit from exploring participants critical thinking on remote PCC. A further limitation is the small study groups. Hence, predictors on meaningful use should be interpreted accordingly as this limited the number of predictors that could be included in the multiple regression models.

## Conclusions

The combined digital platform and structured telephone support could be used as a preventive measure for people diagnosed with COPD or CHF but tends to be more meaningful to use in those diagnosed with COPD. This can be explained by patients with COPD having a greater need for such health care support. Overall, there was a lower level of user adoption of the digital platform compared to telephone support. Additionally, there are shortcomings in the intervention’s implementation that negatively influenced experiences of meaningful use, particularly when the purpose was unclear to patients. Nonetheless, when used, the remote PCC intervention is an easily applicable tool that supports preventive actions in a person-centred manner and can therefore contribute to the provision of equitable care.

## Data Availability

The quantitative dataset generated during and/or analysed during the current study is available from the corresponding author on reasonable request. The dataset generated during individual interviews are not publicly available due to the information provided to the participants when obtaining their informed consent, stating that all attempts would be made to maintain confidentiality. Data is covered by the Public Access to Information and Secrecy act and a confidentiality assessment will be performed at each individual request. Permission from University of Gothenburg, the Institute of Health and Care Science, has to be obtained before data can be accessed. Access could be obtained by contacting Swedish National Data service (SND), University of Gothenburg, Box 463, 405 30 Gothenburg, Sweden. Tel. + 46 31–786 10 00. E-mail: snd@gu.se. Dataset https://doi.org/10.5878/8ycn-k945.

## Abbreviations

aOR: Adjusted odds ratio
AUC: Area under the ROC curve
CHF: Chronic heart failure
CI: Confidence interval
COPD: Chronic obstructive pulmonary disease
OR: Odds ratio
PCC: Person-centred care
PROTECT: Name of the evaluated remote PCC intervention
RCT: Randomised controlled trial
